# The Association between Supraphysiologic Arterial Oxygen Levels and Mortality in Critically Ill Patients. A Multicenter Observational Cohort Study

**DOI:** 10.1164/rccm.201904-0849OC

**Published:** 2019-12-01

**Authors:** Edward Palmer, Benjamin Post, Roman Klapaukh, Giampiero Marra, Niall S. MacCallum, David Brealey, Ari Ercole, Andrew Jones, Simon Ashworth, Peter Watkinson, Richard Beale, Stephen J. Brett, J. Duncan Young, Claire Black, Aasiyah Rashan, Daniel Martin, Mervyn Singer, Steve Harris

**Affiliations:** ^1^ Bloomsbury Institute of Intensive Care Medicine; ^4^ Research Software Development Group, Research IT Services, and; ^5^ Department of Statistical Science, University College London, London, United Kingdom; ^2^ INFORM-lab, London, United Kingdom; ^3^ Department of Critical Care, Barts Health National Health Service (NHS) Trust, London, United Kingdom; ^6^ Department of Critical Care and; ^13^ Therapies and Rehabilitation, University College London Hospitals NHS Foundation Trust, London, United Kingdom; ^7^ Division of Anaesthesia, Department of Medicine, University of Cambridge, Cambridge, United Kingdom; ^8^ Department of Critical Care, Guy’s and St. Thomas’ NHS Foundation Trust, London, United Kingdom; ^9^ Division of Critical Care, Imperial College Healthcare NHS Trust, London, United Kingdom; ^10^ Critical Care Research Group (Kadoorie Centre), Nuffield Department of Clinical Neurosciences, Medical Sciences Division, University of Oxford, Oxford, United Kingdom; ^11^ Centre for Human Applied Physiological Science, King’s College London, London, United Kingdom; ^12^ Department of Surgery and Cancer, Imperial College London, London, United Kingdom; and; ^14^ Division of Surgery and Interventional Science and; ^15^ Critical Care Unit, Royal Free Hospital, London, United Kingdom

**Keywords:** logistic models, critical care, hyperoxia

## Abstract

**Rationale:** There is conflicting evidence on harm related to exposure to supraphysiologic Pa_O_2__ (hyperoxemia) in critically ill patients.

**Objectives:** To examine the association between longitudinal exposure to hyperoxemia and mortality in patients admitted to ICUs in five United Kingdom university hospitals.

**Methods:** A retrospective cohort of ICU admissions between January 31, 2014, and December 31, 2018, from the National Institute of Health Research Critical Care Health Informatics Collaborative was studied. Multivariable logistic regression modeled death in ICU by exposure to hyperoxemia.

**Measurements and Main Results:** Subsets with oxygen exposure windows of 0 to 1, 0 to 3, 0 to 5, and 0 to 7 days were evaluated, capturing 19,515, 10,525, 6,360, and 4,296 patients, respectively. Hyperoxemia dose was defined as the area between the Pa_O_2__ time curve and a boundary of 13.3 kPa (100 mm Hg) divided by the hours of potential exposure (24, 72, 120, or 168 h). An association was found between exposure to hyperoxemia and ICU mortality for exposure windows of 0 to 1 days (odds ratio [OR], 1.15; 95% compatibility interval [CI], 0.95–1.38; *P* = 0.15), 0 to 3 days (OR 1.35; 95% CI, 1.04–1.74; *P* = 0.02), 0 to 5 days (OR, 1.5; 95% CI, 1.07–2.13; *P* = 0.02), and 0 to 7 days (OR, 1.74; 95% CI, 1.11–2.72; *P* = 0.02). However, a dose–response relationship was not observed. There was no evidence to support a differential effect between hyperoxemia and either a respiratory diagnosis or mechanical ventilation.

**Conclusions:** An association between hyperoxemia and mortality was observed in our large, unselected multicenter cohort. The absence of a dose–response relationship weakens causal interpretation. Further experimental research is warranted to elucidate this important question.

**At a Glance Commentary**

**Scientific Knowledge on the Subject**

Oxygen is a drug that carries potential toxicity, but this fact often passes unappreciated in clinical practice. In recent years, hyperoxemia has been increasingly linked with worse outcomes, though the literature is conflicting. Bias may be introduced into studies through confounding by treatment indication, failure to consider oxygen as a longitudinal exposure, and dropout of patients over time.

**What this Study Adds to the Field**

We interrogated a large multicenter cohort of patients requiring at least 24 hours of critical care and used a modelling approach that addressed the above core concerns. We found an association between hyperoxemia and mortality; however, a lack of dose dependency challenges a causal relationship. Our findings support the need for prospective randomized trials with appropriate power.

Oxygen therapy is widely used to treat critically ill patients. British Thoracic Society guidelines regard oxygen as a drug and advise a prescription to accompany its use (1). These guidelines acknowledge potential harm and recommend targeting a specific oxygen saturation range in acutely unwell patients. In adult patients, hyperoxemia may induce hemodynamic changes (2, 3), including vasoconstriction (4, 5), reduced cardiac output, and increased peripheral vascular resistance (6–8); and inflammatory changes, including the generation of reactive oxygen species (9) and absorption atelectasis (10). In healthy subjects, exposure to high inspired oxygen concentrations causes alveolar leak and release of mediators responsible for lung fibrosis (11).

Despite these concerns, other than in patients with type II (hypercarbic) respiratory failure, oxygen use is still largely unregulated in clinical practice. Prospective randomized trials of oxygen therapy in patients suffering myocardial infarction have reported either harm (12, 13) or no effect (14). Increased mortality risk has been suggested in patients receiving higher concentrations of inspired oxygen (15–20) in conditions such as cardiac arrest (21–23) and septic shock (24–26), as well as in general critically ill populations (19, 27). However, most of these studies lack a delineation between harm from appropriately high levels of inspired oxygen used to maintain normoxemia and excessive concentrations that result in hyperoxemia (28). Similarly, analyses of ICU databases variably report an association (29, 30) or lack thereof (31) between hyperoxemia and poor outcomes in the critically ill. Many of these approaches are limited by using only a single measure of Pa_O_2__ or inspired oxygen to define oxygen exposure for an entire ICU admission.

A recent systematic review and meta-analysis of more than 16,000 patients (32) indicated potential harm, concluding, “Patients treated liberally with oxygen had a dose-dependent increased risk of short-term and long-term mortality.” Yet, paradoxically, they could find “no significant difference in disability, hospital-acquired pneumonia, or length of hospital stay.”

The aim of the present study was to determine whether exposure to supraphysiologic Pa_O_2__, measured as time-weighted mean exposure to hyperoxemia (referred to as “hyperoxemia dose” for brevity), was associated with excess ICU mortality. Particular attention was paid to dose–response as a proxy for a causal relationship (33). The specific impact of hyperoxemia was assessed in patients with a primary respiratory diagnosis for ICU admission or those who were mechanically ventilated because concurrent lung inflammation may predispose to pulmonary oxygen toxicity and increased mortality (27).

## Methods

Data were prospectively collected between January 31, 2014, and December 31, 2018, on all adult (≥18 yr) patients attending an ICU from five United Kingdom university hospitals contributing to the National Institute of Health Research Critical Care Health Informatics Collaborative, for which the themes are described elsewhere (34), as is a detailed description of its data specification (35). The legal basis for handling the data is provided in the online supplement. The present study was conducted as a retrospective cohort analysis, with findings reported in accordance with Strengthening the Reporting of Observational Studies in Epidemiology guidance (36).

**Figure 1.**
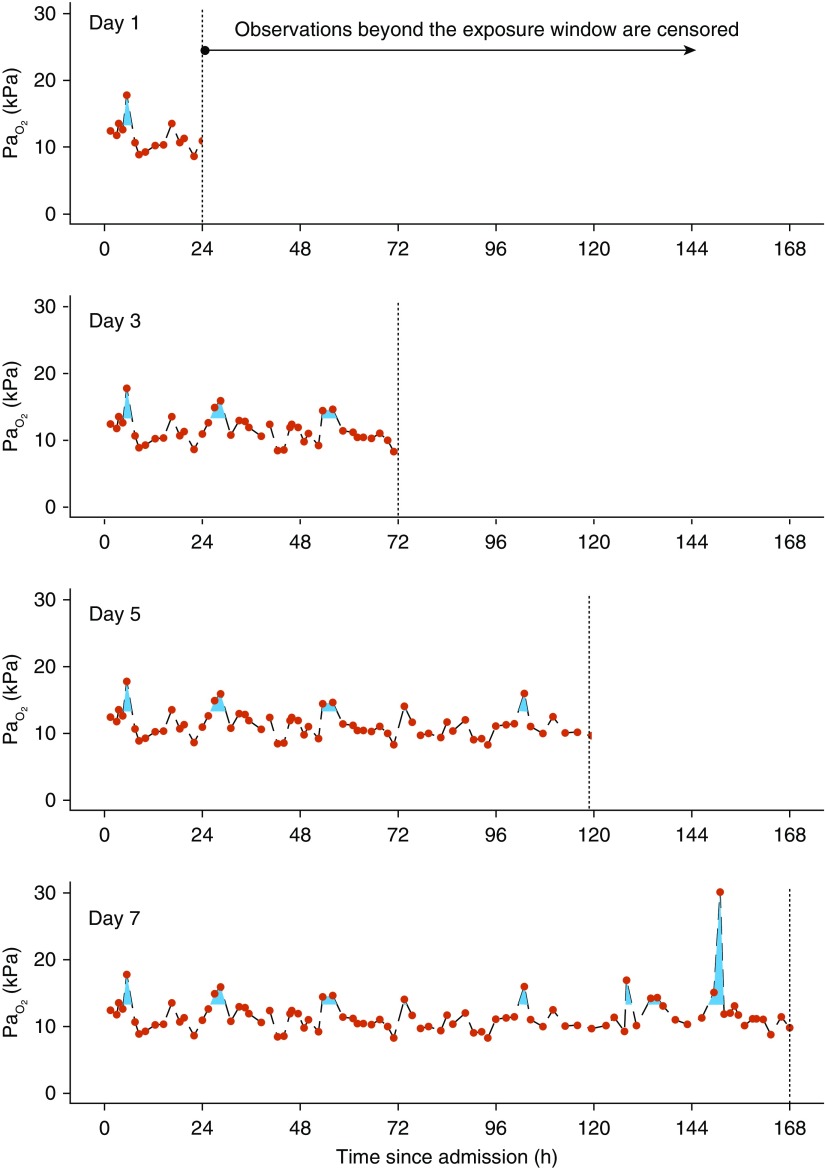
Illustration of the calculation of hyperoxemia dose. The blue area defines hyperoxemia exposure for a real patient drawn from the Critical Care Health Informatics Collaborative database. Red points indicate actual observations. Black interrupted lines show the linear imputation strategy. Gaps exist in the imputation between observations greater than 12 hours apart. Hyperoxemia dose was calculated by summing the blue area and dividing by the hours of the potential exposure window for the given model (from top panel to bottom panel: 24, 72, 120, or 168 h). This yields the natural units originally used to measure Pa_O_2__ (shown in kilopascals). Vertical dashed lines indicate the point of censoring at the end of the exposure window.

Patients were included in the study if their ICU length of stay was longer than 24 hours. Those staying less than 24 hours were typically admitted after elective surgery with very low mortality. These cases were removed because this would lead to prognostic deenrichment while not providing a large enough exposure window for the effects of hyperoxemia to become apparent. Patients with treatment limitation orders, in receipt of cardiopulmonary resucitation in the 24 hours preceeding ICU admission, or failing prespecified data quality checks were excluded. To limit confounding by an unknown exposure to oxygen, or other factors following ICU discharge, only the index admission was considered if a patient had more than one ICU admission. For similar reasons, ICU mortality for that index admission was chosen as the primary endpoint, in preference to hospital mortality or other distant outcome measures. The cohort was narrowed to create nested subsets with progressively longer potential hyperoxemia exposure windows (0–3, 0–5, and 0–7 days). Each subset, therefore, had a period of potential exposure unaffected by informative censoring from either ICU discharge or death (Figure 1; *see* Figure E1 in the online supplement).

ICU mortality was modeled as a function of hyperoxemia dose using multivariable logistic regression. Hyperoxemia dose was defined as the area under the Pa_O_2__ time curve above a threshold Pa_O_2__ value of 13.3 kPa (100 mm Hg) divided by the number of hours of potential exposure. This was applied from the time of ICU admission (Day 0) until 1, 3, 5, or 7 days (Figure 1). Under this definition, 1 kPa (7.6 mm Hg) of hyperoxemia dose describes that a patient’s average Pa_O_2__ was 1 kPa (7.6 mm Hg) above 13.3 kPa (100 mm Hg) for the duration of the exposure window. The 13.3 kPa (100 mm Hg) threshold was chosen because values exceeding this can only be achieved with supplementary oxygen. This boundary, therefore, represents a range of Pa_O_2__ that is unambiguously supraphysiological and hence not confounded by treatment indication.

A substantial proportion of admissions had a hyperoxemia dose of 0. To address this “spike at zero,” an additional covariable indicating any hyperoxemia exposure was added to the model (37). Both covariables (“any hyperoxemia” and hyperoxemia dose) should be considered in concert when interpreting the model.

Other predictor covariables included a primary diagnosis of respiratory illness (yes/no), sex (male/female), age at admission (years), weight (kg), prior need for assisted daily living (independent or any level of dependence), mechanical ventilation for the entirety of the exposure window (yes/no), primary admission reason (medical/surgical), and the Acute Physiology and Chronic Health Evaluation II score. These variables were chosen on the basis of salience to the underlying research question, scientific plausibility, and after exclusion of significant collinearity. Continuous variables were entered without categorization. Age and weight were modeled nonlinearly using restricted cubic splines (38). The Acute Physiology and Chronic Health Evaluation II score was also modeled with restricted cubic splines because evidence from the data supported this decision.

To account for possible differential effects of exposure to hyperoxemia, interaction effects between exposure to hyperoxemia with an underlying respiratory diagnosis and continuous mechanical ventilation were evaluated. Penalized maximum likelihood was applied with a penalty factor determined by optimal model Akaike information criterion (AIC). Penalization was applied to interaction effects only.

Four models were fitted, one for each exposure subset. Figure 1 provides an exemplar case and Figure E1 provides an overview of this process. This procedure was undertaken to balance informative censoring of patient data with the investigation of hyperoxemia, thus maintaining a uniform exposure potential within each subset for this necessarily longitudinal measure.

To create the notion of a continuous time series for Pa_O_2__, which is measured as a point process when arterial blood gas samples are drawn, linear imputation was performed with a 12-hour window. Details of the imputation procedure are presented in Table E1. Where Pa_O_2__ measures were still unavailable, the exposure was assumed to be 0. There were less than 1% missing variables, so a complete case analysis was conducted.

Model validation was performed using bootstrapped corrected calibration plots and Brier scores using 500 resamples. The c-index (area under the receiver operator characteristic curve), precision–recall, and AIC were calculated. The average treatment effect of exposure to hyperoxemia was calculated by fitting models with each individual’s own recorded exposure to hyperoxemia and comparing it with their counterfactual scenario had this exposure been 0.

All statistical analyses were performed using R Version 3.4.4 (R Foundation for Statistical Computing). The full analysis code was made publicly available before manuscript submission (39).

## Results

Over the 4-year period of the study, 45,188 episodes were available. After exclusions, a primary cohort with a minimum 1-day ICU length of stay of 19,515 episodes remained (Figure E2). This cohort was further nested into those who remained in ICU for at least 3 (10,525), 5 (6,360), and 7 (4,296) days. Baseline characteristics for the primary cohort and nested exposure windows are shown in Table 1 and Table E2. A total of 77.5% of patients were exposed to hyperoxemia by Day 1, increasing to 90.6% by Day 7. We observed an association between any hyperoxemia exposure and increased ICU mortality, with an odds ratio (OR) ranging from 1.15 (95% compatibility interval [CI] 0.95–1.38; *P* = 0.15) over Days 0–1 to 1.74 (95% CI, 1.11–2.72; *P* = 0.02) over Days 0–7.

**Table 1.** Abridged Patient Characteristics, Stratified by Nested Exposure Window

Characteristic	1-d Exposure	3-d Exposure	5-d Exposure	7-d Exposure
*n*	19,593	10,571	6,391	4,318
Hyperoxemia dose, kPa	0.54 (0.01–1.75)	0.30 (0.04–0.86)	0.26 (0.04–0.68)	0.27 (0.06–0.65)
Any hyperoxemia exposure (yes)	15,182 (77.5)	8,865 (83.9)	5,580 (87.3)	3,912 (90.6)
Cumulative hyperoxemia exposure, kPa ⋅ h	13.00 (0.4–42.1)	21.84 (2.6–61.6)	31.41 (5.3–81.9)	45.03 (10.6–108.7)
Pre-ICU hospital length of stay, d	1 (1–2)	1 (1–3)	1 (1–3)	1 (1–3)
Age, yr	65 (51–74)	65 (51–75)	64 (49–74)	63 (48–74)
Weight, kg	77 ± 20	77 ± 19	77 ± 20	77 ± 20
Sex				
F	7,834 (40.0)	4,149 (39.2)	2,431 (38.0)	1,621 (37.5)
M	11,758 (60.0)	6,421 (60.7)	3,959 (61.9)	2,696 (62.4)
Not available	1 (0.0)	1 (0.0)	1 (0.0)	1 (0.0)
APACHE II score	15.4 ± 5.8	16.5 ± 6.0	17.2 ± 6.2	17.7 ± 6.3
Prior dependency (none)	16,239 (82.9)	8,575 (81.1)	5,115 (80.0)	3,433 (79.5)
Patient type				
Surgical	10,721 (54.7)	4,652 (44.0)	2,319 (36.3)	1,290 (29.9)
Medical	8,861 (45.2)	5,913 (55.9)	4,067 (63.6)	3,025 (70.1)
Not available	11 (0.1)	6 (0.1)	5 (0.1)	3 (0.1)
Surgical classification				
Elective	6,758 (34.5)	2,597 (24.6)	1,144 (17.9)	539 (12.5)
Scheduled	1,557 (7.9)	804 (7.6)	372 (5.8)	176 (4.1)
Urgent	1,025 (5.2)	412 (3.9)	220 (3.4)	145 (3.4)
Emergency	1,702 (8.7)	1,029 (9.7)	699 (10.9)	517 (12.0)
Not applicable (medical) or not available	8,551 (43.6)	5,729 (54.2)	3,956 (61.9)	2,941 (68.1)
Ethnicity				
Asian/Asian British Indian	335 (1.7)	180 (1.7)	112 (1.8)	85 (2.0)
Asian/Asian British other	335 (1.7)	206 (1.9)	152 (2.4)	119 (2.8)
Black/black British African	555 (2.8)	288 (2.7)	188 (2.9)	128 (3.0)
Black/black British Caribbean	430 (2.2)	211 (2.0)	123 (1.9)	82 (1.9)
Other or not stated	4,746 (24.2)	2,592 (24.5)	1,574 (24.6)	1,031 (23.9)
White British	11,880 (60.6)	6,337 (59.9)	3,759 (58.8)	2,541 (58.8)
White other	1,312 (6.7)	757 (7.2)	483 (7.6)	332 (7.7)
ICU length of stay, d	3.5 (2.0–6.6)	6.0 (4.1–11.0)	9.2 (6.6–16.8)	13.1 (9.1–21.7)
ICU mortality (deceased)	835 (4.3)	577 (5.5)	435 (6.8)	360 (8.3)

*Definition of abbreviation*: APACHE = Acute Physiology and Chronic Health Evaluation.

Variables are presented as mean (SD), median (interquartile range), or count (%) as appropriate. For all characteristics, please *see* Table E1.

**Figure 2.**
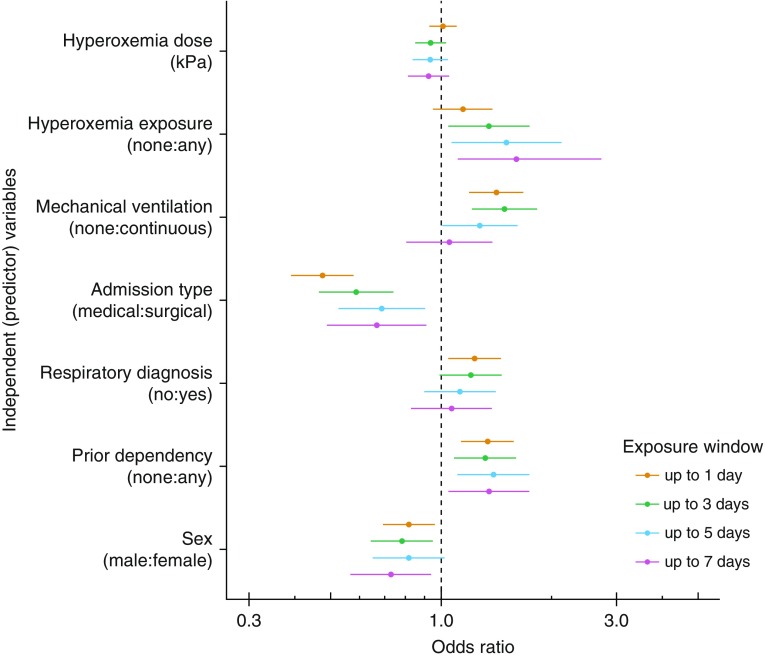
Point estimates of odds ratios and 95% compatibility intervals are presented for all linear model terms. Hyperoxemia has been assessed in two ways: as an indicator (any hyperoxemia exposure) and hyperoxemia dose variables. There was a progressively stronger association between any hyperoxemia exposure and ICU mortality from the Day 0 to Day 1 model up to the Day 0 to Day 7 model. There was a lack of evidence to support a relationship between hyperoxemia dose and ICU mortality. Odds ratios are not presented for age, weight, and the Acute Physiology and Chronic Health Evaluation II score because these were modeled nonlinearly.

There was a lack of evidence to support a dose-dependent effect (Table 2) or the presence of nonlinearities in hyperoxemia dose; accordingly, this component was modeled linearly for parsimony. Point estimates for the ORs and their 95% CIs for covariables are presented in Figure 2. All results are presented in Table E3. These findings were robust to using probit or complementary log–log link functions.

**Table 2.** Odds Ratios (95% Compatibility Intervals) for Hyperoxemia Dose (in Kilopascals) and Any Hyperoxemia Exposure (as Indicator Variable)

Model	Variable	Odds Ratio (95% CI)	Chi Square	DoF	*P* Value
0–1 d	Hyperoxemia dose	1.01 (0.93–1.10)	0.071	1	0.790
	Any hyperoxemia exposure	1.15 (0.95–1.38)	2.110	1	0.146
0–3 d	Hyperoxemia dose	0.94 (0.85–1.03)	1.777	1	0.183
	Any hyperoxemia exposure	1.35 (1.04–1.74)	5.157	1	0.023
0–5 d	Hyperoxemia dose	0.93 (0.83–1.04)	1.441	1	0.230
	Any hyperoxemia exposure	1.5 (1.07–2.13)	5.372	1	0.020
0–7 d	Hyperoxemia dose	0.92 (0.81–1.05)	1.416	1	0.234
	Any hyperoxemia exposure	1.74 (1.11–2.72)	5.815	1	0.016

*Definition of abbreviations*: CI = compatibility interval; DoF = degrees of freedom.

All other predictor variables are described in the online supplement.

There was no overall evidence to support an interaction effect between exposure to hyperoxemia and either an underlying respiratory diagnosis or mechanical ventilation. Likelihood ratios comparing the base model with the penalized maximum likelihood model are shown in Table E4. There was no evidence to support a change in the log odds for death from the interaction between hyperoxemia and either primary respiratory diagnosis or mechanical ventilation status (Table E5). The interaction terms were removed from the final model specification based upon likelihood criteria.

**Figure 3.**
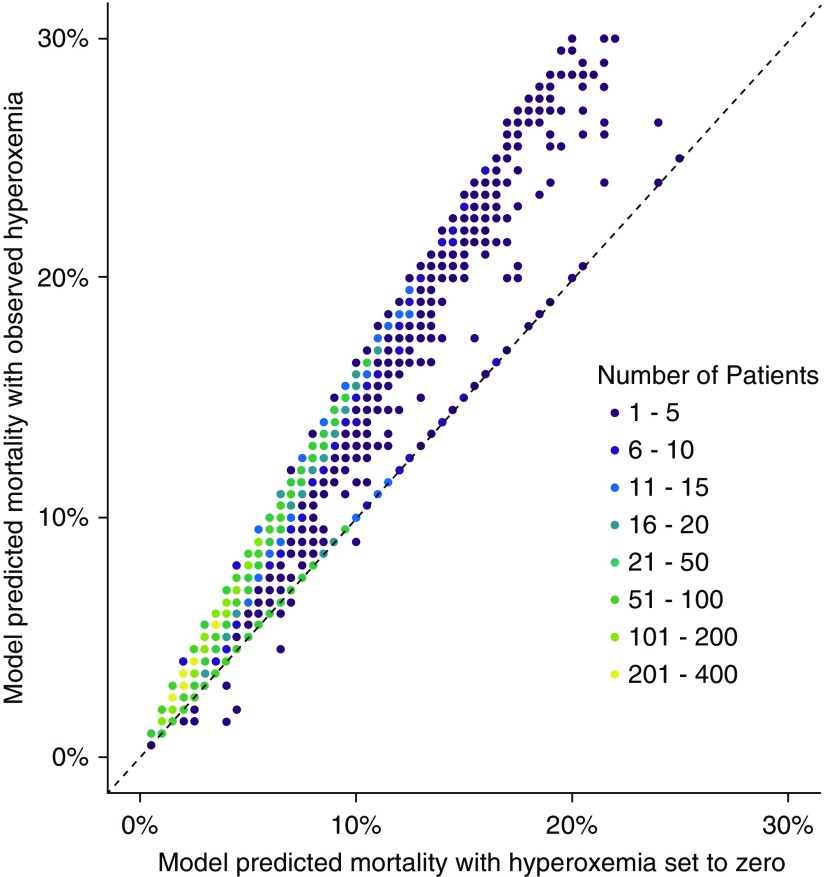
Counterfactual risk plot illustrating the change in predicted mortality by setting all hyperoxemia exposure to 0. The model-predicted risk of mortality with the observed hyperoxemia is shown on the *y*-axis. The model-predicted risk of mortality when setting hyperoxemia to 0 is shown on the *x*-axis. The Day 0 to Day 5 cohort is used as an example (other cohorts demonstrate a similar pattern). The 45° identity line is marked as a dashed diagonal line representing no change in risk. Several observations lie on the identity line, in keeping with the proportion of patients who had no exposure to hyperoxemia and so cannot see an adjustment to their mortality risk via this mechanism.

The modification to risk of mortality between observed exposure to hyperoxemia and the counterfactual scenario setting this exposure to 0 is shown in Figure 3, using the Day 0 to Day 5 cohort as an illustrative example. All models are shown on the absolute risk scale in Figure E3. Point estimates for the average treatment effect were 0.4%, 0.9%, 1.6%, and 2.7% for exposure windows of 0 to 1, 0 to 3, 0 to 5, and 0 to 7, respectively, favoring no exposure to hyperoxemia.

The overall model fit across the four exposure windows was good; each model c-index, optimism-corrected (bootstrapped) Brier score, and AIC is detailed in Table E6. On calibration checks, there was a tendency for models to underpredict mortality in more severe cases (Figure E4).

## Discussion

A consistent association was found across models between any exposure to hyperoxemia for up to 7 days following ICU admission and ICU mortality. This is in keeping with findings from most observational (29, 30, 40, 41) and interventional (17, 18, 27) studies. However, Eastwood and colleagues, using a well controlled model, could not find supporting evidence of an association between hyperoxemia and increased mortality (31).

Crucially, many prior retrospective studies examining the relationship between hyperoxemia and outcome are limited by the availability of longitudinal oxygenation data. A common approach has modeled outcomes as a function of a single arterial blood gas result, usually taken soon after ICU admission. The degree and duration of hyperoxemia before and after this result are undocumented. It is biologically implausible that a single measure of oxygen exposure could shift outcomes so dramatically. Any single measure of oxygenation exposure is likely to be confounded by treatment effects. For example, sicker patients are more likely to be administered higher concentrations of oxygen. This confounding may exert a greater influence on the first arterial blood gas result because it will be this very sample that triggers a de-escalation of oxygen, should this be required.

To our knowledge, only one prior large database study has modeled a longitudinal notion of oxygen (30). The authors found “a dose-response relationship between supra-physiologic arterial oxygen levels and hospital mortality.” Such a hypothesis is difficult to discern, however, given that this effect was only seen in the uppermost category of exposure to oxygen, and a gradient of worsening outcomes across oxygen exposure levels was not demonstrated. Additionally, continuous measures of oxygenation were routinely categorized; this procedure impairs statistical inference, leading to both false-positive findings and reduced statistical power (38). The most directly similar measure in their study to our own approach was a 96-hour area under the curve for Pa_O_2__. This finding was associated with increased hospital (but not ICU) mortality and was found at the upper quintile of exposure only. Under these constraints, there was no clear dose–response relationship. A small study by Ruggiu and colleagues bears a resemblance to our approach in using any Pa_O_2__ greater than or equal to 13.3 kPa (100 mm Hg) to indicate hyperoxemia (40). They modeled mortality with survival analysis and arrived at a similar conclusion that a dose-independent exposure to hyperoxemia was associated with harm. They did not, however, account for informative censoring of patient data.

The varied findings between studies may be due in part to a broad range of oxygenation criteria, statistical methods, and heterogeneous study populations being used to assess the impact of excessive oxygen administration in the ICU (24). Studies have variously used values of oxygenation, including Pa_O_2__ (25, 42, 43), Pa_O_2__ and oxygen saturation as measured by pulse oximetry (Sp_O_2__) (18, 44), Pa_O_2__ and Fi_O_2__ (29, 41), and alveolar-arteriolar oxygen gradient (31). From a biological standpoint, it remains unclear which of these (or combination thereof) provides the best measure to elucidate harm. Sp_O_2__ has a ceiling effect at 100% and so is limited in its capacity to reveal excess oxygenation. The relationship between Sp_O_2__ and Pa_O_2__ may be altered by pathophysiology and ageing (45). Fi_O_2__ is strongly confounded with a treatment effect because patients with high Fi_O_2__ requirements are more likely to have higher disease severity (46, 47). Our approach has the merit of using longitudinal information regarding the arterial oxygenation status of each patient throughout the study period. By calculating the hyperoxemia dose and accounting for the effect of spike at zero (37), questions relating to a dose–response relationship can be addressed in a principled manner. This approach may better explain systematic variance in outcomes above what could be achieved by previously reported strategies.

We were unable to find supporting evidence for a dose–response relationship between hyperoxemia dose and ICU mortality. This does not necessarily mean that this effect is absent; however, this weakens causal interpretation of our findings. A cut point of 13.3 kPa (100 mm Hg) was used to define hyperoxemia, rather than modeling the entire area under the Pa_O_2__ time curve. This latter approach would lead to inescapable unmeasured confounding by severity of illness that can prove challenging to adequately control for. In our experience, longitudinal measures of acute illness severity, particularly those that encompass a notion of respiratory dysfunction, are particularly volatile. Our definition makes minimal assumptions about what constitutes hyperoxemia but at the expense of reducing the number of cases from which to learn. Given the reducing number of cases without exposure to hyperoxemia, particularly toward 7 days, residual confounding remains a concern as a potential explanation of these findings.

There was no evidence to support the presence of a differential effect of exposure to hyperoxemia regardless of primary respiratory diagnosis or mechanical ventilation status. There may, however, have been inadequate power in our cohort to detect these effects.

In terms of limitations, we conducted a two-stage analysis of longitudinal data. In this approach, a longitudinal process, such as serial Pa_O_2__, is collapsed into a single measure to be included within a model. Although this is a common approach, there is necessarily a loss of statistical information. We are thus unable to address questions related to, for example, the profile of oxygen exposure over an ICU admission. Under our approach, exposure to high levels of excess oxygen for a short period of time are thought of as equal to low levels of excess oxygen for a long period of time.

We sought to apply a methodologically rigorous approach to this problem, reducing the bias inherent in studies of this nature by accounting for informative censoring, exploring dose–response relationships and interaction effects. Nevertheless, the associations described could still represent particular patient subgroups known to experience higher mortality and regular exposure to hyperoxemia; for example, those who undergo multiple transfers and procedures. These patients are inherently less stable, experience higher mortality (48) and morbidity (49), and may be placed on a high inspired oxygen concentration for transfer, regardless of clinical need. Such events are common and our model would highlight these associations.

There is likely a large and variable exposure to oxygen before ICU admission. Information with regard to oxygenation of patients outside the ICU was unavailable in our database. Given that patients from our cohort enter critical care after variable amounts of time in an operating room, emergency department, or ward, it is reasonable to assume that most have had a prior exposure to oxygen. Indeed, even if normoxemia is achieved after admission to ICU, a brief period of hyperoxemia in the emergency department has been suggested to be detrimental (50). Should exposure to hyperoxemia increase the risk of mortality, it is unclear over what timeframe following exposure this risk returns to baseline. We chose to model ICU mortality in place of other more distant measures of outcome (hospital mortality, 90-day mortality etc.) because the proximity of the outcome to our measure of oxygen exposure helps to elucidate a causal relationship, if one exists. We chose to censor readmissions from the model for similar reasoning because this would induce a large unaccounted-for exposure to oxygen between admissions.

We chose to model a function of Pa_O_2__ (hyperoxemia dose) because this approach implicitly addresses the problem of confounding by treatment effect, albeit at the expense of creating an imperfect definition of excess oxygen exposure. A Pa_O_2__ above 13.3 kPa (100 mm Hg) likely captures a surrogate of the mechanism that is causing harm (high inspired oxygen concentrations). Much of the preclinical data favors high Fi_O_2__ as being causative for lung parenchymal damage (9). However, there may be other unrecognized systemic effects that result from supraphysiological Pa_O_2__.

We did not model Pa_O_2__ directly because this holds a nonmonotonic relationship with mortality; hypoxemia and hyperoxemia are both thought to be detrimental (51, 52). Thus, by constraining this variable as hyperoxemia dose, we could investigate the effect of hyperoxemia, without needing to account for hypoxemia, and thus create a more parsimonious model.

Exposure to hyperoxemia is an inherently time-dependent variable. As such, it is difficult to model this phenomenon inside the ICU for two main reasons. First, informative censoring will bias results (patients get better or die, and stop contributing data at variable nonrandom points in time). Second, to measure hyperoxemia dose, a window of observation is required to demonstrate an effect. We tested over several time windows to balance the tension between patient numbers and the opportunity for hyperoxemia exposure.

### Conclusions

This study suggests that exposure to supraphysiologic levels of oxygen is associated with harm in the critically ill patient. We were, however, unable to find evidence supporting a dose–response relationship between exposure to supraphysiologic oxygenation and mortality. The lack of a dose–response relationship weakens any causal interpretation of this finding or implies that the effect is relatively small and/or reaches a plateau. We cannot, however, exclude an undetected dose-dependent effect. Placing these findings within the context of the broader literature, our study suggests that a small but meaningful reduction in mortality could be achieved by avoiding exposure to hyperoxemia. However, the potential for unmeasured confounding to bias this result places strong caveats on a causal interpretation. Further experimental investigation into this controversial field is thus warranted.

## References

[1] O’Driscoll BR Howard LS Earis J Mak V BTS guideline for oxygen use in adults in healthcare and emergency settings *Thorax* 2017 72 ii1 ii90 10.1136/thoraxjnl-2016-209729 28507176

[2] Helmerhorst HJF de Wilde RBP Lee DH Palmen M Jansen JRC van Westerloo DJ *et al* Hemodynamic effects of short-term hyperoxia after coronary artery bypass grafting *Ann Intensive Care* 2017 7 20 28233196 10.1186/s13613-017-0246-9 PMC5323416

[3] Bak Z Sjöberg F Rousseau A Steinvall I Janerot-Sjoberg B Human cardiovascular dose-response to supplemental oxygen *Acta Physiol (Oxf)* 2007 191 15 24 17506865 10.1111/j.1748-1716.2007.01710.x

[4] McNulty PH Robertson BJ Tulli MA Hess J Harach LA Scott S *et al* Effect of hyperoxia and vitamin C on coronary blood flow in patients with ischemic heart disease *J Appl Physiol (1985)* 2007 102 2040 2045 17303710 10.1152/japplphysiol.00595.2006

[5] Dollery CT Hill DW Mailer CM Ramalho PS High oxygen pressure and the retinal blood-vessels *Lancet* 1964 2 291 292 14163671 10.1016/s0140-6736(64)93051-x

[6] Haque WA Boehmer J Clemson BS Leuenberger UA Silber DH Sinoway LI Hemodynamic effects of supplemental oxygen administration in congestive heart failure *J Am Coll Cardiol* 1996 27 353 357 8557905 10.1016/0735-1097(95)00474-2

[7] Milone SD Newton GE Parker JD Hemodynamic and biochemical effects of 100% oxygen breathing in humans *Can J Physiol Pharmacol* 1999 77 124 130 10535703

[8] Ganz W Donoso R Marcus H Swan HJ Coronary hemodynamics and myocardial oxygen metabolism during oxygen breathing in patients with and without coronary artery disease *Circulation* 1972 45 763 768 5016013 10.1161/01.cir.45.4.763

[9] Shimada I Kubota A Katoh M Suzuki F Hyperoxia causes diffuse alveolar damage through mechanisms involving upregulation of c-Myc/Bax and enhanced production of reactive oxygen species *Respir Investig* 2016 54 59 68 10.1016/j.resinv.2015.08.006 26718146

[10] Aboab J Jonson B Kouatchet A Taille S Niklason L Brochard L Effect of inspired oxygen fraction on alveolar derecruitment in acute respiratory distress syndrome *Intensive Care Med* 2006 32 1979 1986 17019545 10.1007/s00134-006-0382-4

[11] Davis WB Rennard SI Bitterman PB Crystal RG Pulmonary oxygen toxicity: early reversible changes in human alveolar structures induced by hyperoxia *N Engl J Med* 1983 309 878 883 6888481 10.1056/NEJM198310133091502

[12] Stub D Smith K Bernard S Nehme Z Stephenson M Bray JE *et al* AVOID Investigators Air versus oxygen in ST-segment-elevation myocardial infarction *Circulation* 2015 131 2143 2150 26002889 10.1161/CIRCULATIONAHA.114.014494

[13] Wijesinghe M Perrin K Ranchord A Simmonds M Weatherall M Beasley R Routine use of oxygen in the treatment of myocardial infarction: systematic review *Heart* 2009 95 198 202 18708420 10.1136/hrt.2008.148742

[14] Hofmann R James SK Jernberg T Lindahl B Erlinge D Witt N *et al* DETO2X–SWEDEHEART Investigators Oxygen therapy in suspected acute myocardial infarction *N Engl J Med* 2017 377 1240 1249 28844200 10.1056/NEJMoa1706222

[15] Damiani E Adrario E Girardis M Romano R Pelaia P Singer M *et al* Arterial hyperoxia and mortality in critically ill patients: a systematic review and meta-analysis *Crit Care* 2014 18 711 25532567 10.1186/s13054-014-0711-x PMC4298955

[16] Eastwood GM Tanaka A Espinoza EDV Peck L Young H Mårtensson J *et al* Conservative oxygen therapy in mechanically ventilated patients following cardiac arrest: a retrospective nested cohort study *Resuscitation* 2016 101 108 114 26718090 10.1016/j.resuscitation.2015.11.026

[17] Suzuki S Eastwood GM Glassford NJ Peck L Young H Garcia-Alvarez M *et al* Conservative oxygen therapy in mechanically ventilated patients: a pilot before-and-after trial *Crit Care Med* 2014 42 1414 1422 24561566 10.1097/CCM.0000000000000219

[18] Helmerhorst HJF Schultz MJ van der Voort PHJ Bosman RJ Juffermans NP de Wilde RBP *et al* Effectiveness and clinical outcomes of a two-step implementation of conservative oxygenation targets in critically ill patients: a before and after trial *Crit Care Med* 2016 44 554 563 26562347 10.1097/CCM.0000000000001461

[19] Girardis M Busani S Damiani E Donati A Rinaldi L Marudi A *et al* Effect of conservative vs conventional oxygen therapy on mortality among patients in an intensive care unit: the oxygen-ICU randomized clinical trial *JAMA* 2016 316 1583 1589 27706466 10.1001/jama.2016.11993

[20] Helmerhorst HJF Roos-Blom M-J van Westerloo DJ de Jonge E Association between arterial hyperoxia and outcome in subsets of critical illness: a systematic review, meta-analysis, and meta-regression of cohort studies *Crit Care Med* 2015 43 1508 1519 25855899 10.1097/CCM.0000000000000998

[21] Kilgannon JH Jones AE Shapiro NI Angelos MG Milcarek B Hunter K *et al* Emergency Medicine Shock Research Network (EMShockNet) Investigators Association between arterial hyperoxia following resuscitation from cardiac arrest and in-hospital mortality *JAMA* 2010 303 2165 2171 20516417 10.1001/jama.2010.707

[22] Kilgannon JH Jones AE Parrillo JE Dellinger RP Milcarek B Hunter K *et al* Emergency Medicine Shock Research Network (EMShockNet) Investigators Relationship between supranormal oxygen tension and outcome after resuscitation from cardiac arrest *Circulation* 2011 123 2717 2722 21606393 10.1161/CIRCULATIONAHA.110.001016

[23] Roberts BW Kilgannon JH Hunter BR Puskarich MA Pierce L Donnino M *et al* Association between early hyperoxia exposure after resuscitation from cardiac arrest and neurological disability: prospective multicenter protocol-directed cohort study *Circulation* 2018 137 2114 2124 29437118 10.1161/CIRCULATIONAHA.117.032054 PMC6370332

[24] Asfar P Schortgen F Boisramé-Helms J Charpentier J Guérot E Megarbane B *et al* HYPER2S Investigators REVA research network Hyperoxia and hypertonic saline in patients with septic shock (HYPERS2S): a two-by-two factorial, multicentre, randomised, clinical trial *Lancet Respir Med* 2017 5 180 190 28219612 10.1016/S2213-2600(17)30046-2

[25] Stolmeijer R ter Maaten JC Zijlstra JG Ligtenberg JJM Oxygen therapy for sepsis patients in the emergency department: a little less? *Eur J Emerg Med* 2014 21 233 235 23611817 10.1097/MEJ.0b013e328361c6c7

[26] Demiselle J Wepler M Hartmann C Radermacher P Schortgen F Meziani F *et al* HYPER2S investigators Hyperoxia toxicity in septic shock patients according to the Sepsis-3 criteria: a post hoc analysis of the HYPER2S trial *Ann Intensive Care* 2018 8 90 30225670 10.1186/s13613-018-0435-1 PMC6141409

[27] Panwar R Hardie M Bellomo R Barrot L Eastwood GM Young PJ *et al* CLOSE Study Investigators ANZICS Clinical Trials Group Conservative versus liberal oxygenation targets for mechanically ventilated patients: a pilot multicenter randomized controlled trial *Am J Respir Crit Care Med* 2016 193 43 51 26334785 10.1164/rccm.201505-1019OC

[28] Sjöberg F Singer M The medical use of oxygen: a time for critical reappraisal *J Intern Med* 2013 274 505 528 24206183 10.1111/joim.12139

[29] de Jonge E Peelen L Keijzers PJ Joore H de Lange D van der Voort PHJ *et al* Association between administered oxygen, arterial partial oxygen pressure and mortality in mechanically ventilated intensive care unit patients *Crit Care* 2008 12 R156 19077208 10.1186/cc7150 PMC2646321

[30] Helmerhorst HJF Arts DL Schultz MJ van der Voort PHJ Abu-Hanna A de Jonge E *et al* Metrics of arterial hyperoxia and associated outcomes in critical care *Crit Care Med* 2017 45 187 195 27763912 10.1097/CCM.0000000000002084

[31] Eastwood G Bellomo R Bailey M Taori G Pilcher D Young P *et al* Arterial oxygen tension and mortality in mechanically ventilated patients *Intensive Care Med* 2012 38 91 98 22127482 10.1007/s00134-011-2419-6

[32] Chu DK Kim LH-y Young PJ Zamiri N Almenawer SA Jaeschke R *et al* Mortality and morbidity in acutely ill adults treated with liberal versus conservative oxygen therapy (IOTA): a systematic review and meta-analysis *Lancet* 2018 391 1693 1705 29726345 10.1016/S0140-6736(18)30479-3

[33] Hill AB The environment and disease: association or causation? *Proc R Soc Med* 1965 58 295 300 14283879 10.1177/003591576505800503 PMC1898525

[34] NIHR health data finder 2018 [accessed 2019 Jul 1]. Available from http://www.hdf.nihr.ac.uk

[35] Harris S Shi S Brealey D MacCallum NS Denaxas S Perez-Suarez D *et al* Critical Care Health Informatics Collaborative (CCHIC): data, tools and methods for reproducible research. A multi-centre UK intensive care database *Int J Med Inform* 2018 112 82 89 29500026 10.1016/j.ijmedinf.2018.01.006

[36] von Elm E Altman DG Egger M Pocock SJ Gøtzsche PC Vandenbroucke JP STROBE Initiative The Strengthening the Reporting of Observational Studies in Epidemiology (STROBE) statement: guidelines for reporting observational studies *Lancet* 2007 370 1453 1457 18064739 10.1016/S0140-6736(07)61602-X

[37] Royston P Sauerbrei W Becher H Modelling continuous exposures with a ‘spike’ at zero: a new procedure based on fractional polynomials *Stat Med* 2010 29 1219 1227 20191601 10.1002/sim.3864

[38] Harrell F Regression modeling strategies with applications to linear models, logistic and ordinal regression, and survival analysis New York, NY Springer 2015

[39] Palmer E CC-HIC/hyperoxaemia: Post peer review release (version 1.2). Zenodo. 2019 Jul 11 [accessed 2019 Oct 30]. Available from: 10.5281/zenodo.3332775

[40] Ruggiu M Aissaoui N Nael J Haw-Berlemont C Herrmann B Augy J-L *et al* Hyperoxia effects on intensive care unit mortality: a retrospective pragmatic cohort study *Crit Care* 2018 22 218 30236126 10.1186/s13054-018-2142-6 PMC6148961

[41] Rachmale S Li G Wilson G Malinchoc M Gajic O Practice of excessive F_IO_2__ and effect on pulmonary outcomes in mechanically ventilated patients with acute lung injury *Respir Care* 2012 67 1887 1893 10.4187/respcare.01696 22613692

[42] Elmer J Scutella M Pullalarevu R Wang B Vaghasia N Trzeciak S *et al* Pittsburgh Post-Cardiac Arrest Service (PCAS) The association between hyperoxia and patient outcomes after cardiac arrest: analysis of a high-resolution database *Intensive Care Med* 2015 41 49 57 25472570 10.1007/s00134-014-3555-6 PMC4337386

[43] Vaahersalo J Bendel S Reinikainen M Kurola J Tiainen M Raj R *et al* FINNRESUSCI Study Group Arterial blood gas tensions after resuscitation from out-of-hospital cardiac arrest: associations with long-term neurologic outcome *Crit Care Med* 2014 42 1463 1470 24557423 10.1097/CCM.0000000000000228

[44] Durlinger EMJ Spoelstra-de Man AME Smit B de Grooth HJ Girbes ARJ Oudemans-van Straaten HM *et al* Hyperoxia: at what level of SpO_2_ is a patient safe? A study in mechanically ventilated ICU patients *J Crit Care* 2017 39 199 204 28279497 10.1016/j.jcrc.2017.02.031

[45] Martin DS Levett DZH Grocott MPW Montgomery HE Variation in human performance in the hypoxic mountain environment *Exp Physiol* 2010 95 463 470 19946029 10.1113/expphysiol.2009.047589

[46] Pisani L Roozeman J-P Simonis FD Giangregorio A van der Hoeven SM Schouten LR *et al* MARS consortium Risk stratification using SpO_2_/FiO_2_ and PEEP at initial ARDS diagnosis and after 24 h in patients with moderate or severe ARDS *Ann Intensive Care* 2017 7 108 29071429 10.1186/s13613-017-0327-9 PMC5656507

[47] Adams JY Rogers A Schuler A Marelich GP Fresco JM Taylor SL *et al* The association between SpO2/FiO2 ratio time-at-risk and hospital mortality in mechanically ventilated patients *Am J Respir Crit Care Med* 2017 195 A5029 10.7812/TPP/19.113 PMC7021142 32069205

[48] Beckmann U Gillies DM Berenholtz SM Wu AW Pronovost P Incidents relating to the intra-hospital transfer of critically ill patients: an analysis of the reports submitted to the Australian Incident Monitoring Study in Intensive Care *Intensive Care Med* 2004 30 1579 1585 14991102 10.1007/s00134-004-2177-9

[49] Papson JPN Russell KL Taylor DM Unexpected events during the intrahospital transport of critically ill patients *Acad Emerg Med* 2007 14 574 577 17535981 10.1197/j.aem.2007.02.034

[50] Page D Ablordeppey E Wessman BT Mohr NM Trzeciak S Kollef MH *et al* Emergency department hyperoxia is associated with increased mortality in mechanically ventilated patients: a cohort study *Crit Care* 2018 22 9 29347982 10.1186/s13054-017-1926-4 PMC5774130

[51] Lee BK Jeung KW Lee HY Lee SJ Jung YH Lee WK *et al* Association between mean arterial blood gas tension and outcome in cardiac arrest patients treated with therapeutic hypothermia *Am J Emerg Med* 2014 32 55 60 24210887 10.1016/j.ajem.2013.09.044

[52] Davis DP Meade W Sise MJ Kennedy F Simon F Tominaga G *et al* Both hypoxemia and extreme hyperoxemia may be detrimental in patients with severe traumatic brain injury *J Neurotrauma* 2009 26 2217 2223 19811093 10.1089/neu.2009.0940

